# Indoor Air Nicotine, Nitrogen Dioxide Exposure, and COVID-19 Outcomes in a Great Plains Tribal Community

**DOI:** 10.1155/ina/7749725

**Published:** 2026-03-31

**Authors:** Anna Tillery, Rae O’Leary, Nora Pisanic, Brittany Youpee, Angela Aherrera, Kate Krucynski, Jaylynn Farlee, Megan Wilks, Erena S. Hovhannisyan Romero, Miranda Cajero, Jacob Duran, Christopher Heaney, Ana M. Rule, Esther Erdei

**Affiliations:** 1 Environmental Health and Engineering Department, Johns Hopkins University Bloomberg School of Public Health, Baltimore, Maryland, USA; 2 Environmental Health Sciences Department, Columbia University Mailman School of Public Health, New York, New York, USA; 3 Missouri Breaks Industries Research Inc., Eagle Butte, South Dakota, USA; 4 Department of Biostatistics, Epidemiology and Informatics, University of Pennsylvania Perelman School of Medicine, Philadelphia, Pennsylvania, USA; 5 Department of Pharmaceutical Sciences, The University of New Mexico Health Sciences Center College of Pharmacy, Albuquerque, New Mexico, USA

**Keywords:** air pollution, community-engaged research, COVID-19, indoor air, Tribal communities

## Abstract

**Introduction:**

Indigenous communities in the United States have disproportionately high tobacco use and reliance on propane for heating and cooking fuel compared to other demographic groups. Of concern is exposure to air nicotine (AN) and NO_2_ in indoor environments and their potential impact on respiratory infections.

**Objective:**

The objective of this study is to assess indoor air pollution and COVID-19 infection outcomes in multigenerational households within the Cheyenne River Sioux Tribe (CRST).

**Methods:**

The COVID-19 Wayakta He? (“Are you on guard against COVID-19?”) Study recruited 290 multigenerational households with two adults at least 9 years apart. All participants responded to a questionnaire on COVID-19 outcomes, housing conditions, and environmental health factors. Households deployed passive AN and NO_2_ air monitors in their homes for 1 week. Each participant provided saliva samples for COVID-19 serological testing.

**Results:**

The geometric mean AN concentration was 0.052 *μ*g/m^3^ (range: 0.017–5.35); 40.1% of households had AN detected. The geometric mean NO_2_ concentration was 10.5 ppb (range: 0.65–138). The odds of previous COVID-19 infection were 1.21 (95% CI: 1.01, 1.51) times higher for those exposed to AN; the odds of self-reported long COVID-19 disease were 3.69 (95% CI: 1.11, 16.6) times higher for those exposed to NO_2_.

**Conclusion:**

Indoor air exposures to AN and NO_2_ increased the odds of having COVID-19 infection and long COVID-19 outcomes. The combination of toxic chemical exposures, increased prevalence of existing chronic diseases, and structural social problems placed Indigenous communities at increased risk of having COVID-19 infections. High exposures to household AN and NO_2_ and the occurrence of COVID-19 disease highlight the importance of consistently including exposure assessments to design targeted interventions in Tribal communities.

## 1. Introduction

It has been documented for decades that American Indian and Alaskan Native (AI/AN) Tribal members experience an increased burden from infectious agents [1–3]. This health concern was further deepened during the COVID-19 (SARS-CoV-2 viral infection) pandemic [4, 5]. During the early months of the pandemic, before vaccinations were available, AI/AN people had the greatest rates of COVID-19-associated hospitalizations and deaths compared to other racial or ethnic groups [6–9]. The divergence in COVID-19 health outcomes among AI/AN people demonstrated an immediate need to support Tribal public health responses in the struggle with SARS-CoV-2 infections.

The unique cultures of Indigenous communities in the Great Plains, coupled with the historical exclusion of AI/AN people from research, highlight the importance of designing culturally appropriate and community-based exposure assessments that support community health interventions [10]. This was particularly important during the shelter-in-place policies implemented during the COVID-19 pandemic, as more people stayed home and were exposed to indoor air contaminants [11–13].

The high prevalence of tobacco use among Great Plains Native communities (approximately 50% of adults in some areas) means that exposure to AN is common [13, 14]. Additionally, severe winters and remote home locations require these communities to heavily rely on propane for fuel, which can result in NO_2_ exposures [12, 15]. Exposure to AN through secondhand smoke and electronic cigarette (e-cigarette) aerosols has been shown to diminish the body’s immune response to viral infections and increase the risk of COVID-19 infection [15, 16]. Exposures to NO_2_ have also been shown to exacerbate upper respiratory infections and worsen health outcomes, especially in vulnerable populations such as children and the elderly [17–19]. Thus, both AN and NO_2_ have the potential to worsen SARS-CoV-2 viral infections and may have compounded the impact of an already devastating pandemic on AI/AN people.

The Cheyenne River Sioux Tribe (CRST) Reservation, located in the state of South Dakota, was particularly suited to study the effects of indoor air on respiratory infections due to their decades-long relationship with the academic research community. The CRST has partnered with the local Missouri Breaks Industries Research Inc. (MBIRI) to investigate Tribal public health concerns and design targeted policies to address them. With help from MBIRI, the CRST has mounted legal efforts to combat predatory advertising conducted by tobacco companies and championed secondhand smoke initiatives to reduce AN in public spaces [13, 20]. Throughout the COVID-19 pandemic, the tribe implemented a robust public health response, initially limiting travel across the reservation borders and later investing in incentives for completion of the vaccination series against SARS-CoV-2 [11, 21, 22]. The CRST COVID-19 Wayakta He? (“Are you on guard against COVID-19?”) Study, in partnership between MBIRI, Johns Hopkins University, and the University of New Mexico Health Sciences Center, was created to investigate the relationships between indoor air exposures, nicotine use, and social stressors on immunological responses and COVID-19 disease outcomes.

The objective of this study is to assess the impact that the indoor air environment has on COVID-19 disease in this rural, AI/AN community. We hypothesized that increased SARS-CoV-2 infection and more severe outcomes among CRST community members were associated with secondhand smoke and propane fuel use in homes, lower socioeconomic status, and large multigenerational households. It is currently unknown what effect these community attributes had on the COVID-19 pandemic in the CRST, and no other Tribal work focused on these complex problems has been carried out. To test the hypotheses, we assessed the relationship between indoor air contaminants, self-reported questionnaire data, and salivary antibodies indicative of SARS-CoV-2 infection. Understanding these relationships will further assist Tribal leadership in preparing for future public health crises.

## 2. Methods

### 2.1. Study Population and Recruitment

We successfully recruited 290 multigenerational households targeting all 23 local communities in the CRST. The goal was to recruit two adults per household who were aged at least 9 years apart between February 2022 and May 2023. Recruitment tactics and methods were documented previously by Tillery et al. [14].

Each of the 290 households received a package that contained two questionnaires and an AN air sampler. Due to limited resources, in 100 of the homes, packages contained an additional sampler for NO_2_ monitoring. Ten percent of packages contained a second monitor labeled “duplicate” and/or a second “blank” monitor for quality control. MBIRI staff provided written, video, and verbal instructions for each step of sampling. Instructions on how to return samples and surveys were given by MBIRI staff during enrollment and before drop-off. One participant was designated the head of household (HOH); their questionnaire contained additional questions on household conditions and environment. When participants returned samples and surveys, research staff collected passive drool saliva and gingival crevicular fluid from both participating household members. The study protocol was approved by the Great Plains Institutional Review Board (IRB) #21-R-23GP, the IRBs at the University of New Mexico #21–359 (Albuquerque, New Mexico), and through a reliance agreement by Johns Hopkins University (Baltimore, Maryland). All participants provided written informed consent and agreed to biospecimen collection and storage.

### 2.2. Patient and Public Involvement

The CRST Tribal Council and the public were involved in the design and conduct of this study. In May 2020, a letter of support was received from the CRST Tribal Council Chairman. During the creation and planning of the study, MBIRI presented the study design to both the CRST Tribal Council and a Missouri Breaks Community Advisory Group for feedback and approval in the fall of 2021. Community members and Tribal Council representatives requested long COVID-19 outcomes in data collection, and as a result, questions regarding these outcomes were added to our survey. Tribal members and current participants were encouraged to share this study with friends and family to increase recruitment. Dissemination materials, including individual results, aggregate infographics, radio interviews, videos, and oral presentations, were shared with the community at large and Community Advisory Group members.

### 2.3. Air Sampling and Analysis

The HOH was instructed to set up monitors and record the date and time in the provided log sheet. The monitors were deployed for 1 week, after which the participants were instructed to take them down, record the date and time, and return to MBIRI. Both types of monitors (AN and NO_2_) had a clip built in for easy hanging out of reach of children in common areas of the home. The AN and NO_2_ filters were shipped to Johns Hopkins University (Baltimore, Maryland) and stored under refrigeration (4°C) until analysis.

Vapor-phase nicotine was collected by passive, diffusion-based samplers containing a filter treated with sodium bisulfate [23]. The AN filters were extracted with an internal standard (Nicotine-d3; Supelco, Bellefonte, Pennsylvania, United States) and analyzed with a 5-point calibration curve using gas chromatography with a nitrogen phosphorous detector (NPD) (GC-2014/NPD; Shimadzu, Kyoto, Japan). Airborne nicotine concentrations were calculated by dividing the amount of nicotine collected on each filter (mg) by the volume of air sampled (m^3^), equal to the total sampling time multiplied by the effective flow rate (25 mL/min).

We measured NO_2_ by passive, diffusion-based samplers (Ogawa & Co, Pompano Beach, Florida, United States). One day (24 h) before analysis, a sulfanilamide solution and 0.56 g *N*-(1-naphthyl)-ethylenediamine dihydrochloride (NEDA) were prepared and refrigerated (4°C). On the day of analysis, NO_2_ filters were placed in 8 mL of deionized water to produce nitrite ions. Each filter was treated with 2 mL of the sulfanilamide and NEDA solutions. The treated filters were analyzed with a spectrophotometer at a wavelength of 545 nm. Three lab-prepared blanks and six standard solutions (0–1.0 *μ*g nitrite/mL) were analyzed with the samples to calculate a standard curve. Absorbance values from the spectrophotometer were divided by the slope of the standard curve to determine the collected mass (ng) of nitrite. The NO_2_ concentration was determined by multiplying the mass by a temperature- and humidity-dependent conversion coefficient (*α*NO_2_) and dividing by the volume of air. All samples that were below the limit of detection (LOD) (AN = 0.034 *μ*g/m^3^; NO_2_ = 2.5 ppb) were replaced with the LOD divided by the square root of two.

### 2.4. Biospecimen Collection and Analysis

After air samples had been deployed for 7 days, research staff collected participants’ saliva samples at the research clinic or the participants’ homes. Two saliva samples were collected from each participant: (1) a gingival crevicular oral fluid sample (Oracol+ saliva collection swab, Malvern Medical Developments, Worcester, United Kingdom), to test for the presence of salivary SARS-CoV-2 IgG, and (2) a passive drool sample to test active SARS-CoV-2 viral infection. For the Oracol+ sample, the participant held the handle, inserted the sponge between their gums and cheek at the gum line, and rubbed the sponge against their gums for at least 1 min, until the sponge was saturated. Passive drool was collected by participant pooling saliva in their mouth and letting it fall into the saliva collection tube. If collection occurred at the participant’s home, samples were stored in a cold box with ice packs and transported back to MBIRI. Samples were shipped on ice to Johns Hopkins University (Baltimore, Maryland) within 14 days of collection. Samples were refrigerated (4°C) until processed in the lab.

## 3. Analysis

### 3.1. Active SARS-CoV-2 Infection Status

Active SARS-CoV-2 infection status was measured using the Food and Drug Administration (FDA) Emergency Use Authorization SalivaDirect method (SalivaDirect Inc, New Haven, United States) at Johns Hopkins University (Baltimore, Maryland) [24]. Briefly, 50 *μ*L of homogenized passive drool was digested with 2.5 *μ*L proteinase K (50 *μ*g/mL, Thermo Fisher Scientific). Samples were mixed, spun down, and heat-inactivated. Each 20 *μ*L PCR reaction contained 100 nM N1 and RNAse P primers and 100 nM probe, 10 *μ*L Luna 2x RT-PCR mix (New England Biolabs, Ipswich, Massachusetts, United States), and 5 *μ*L template (passive drool) or controls (nontemplate [water] control or Twist Synthetic SARS-CoV-2 RNA at 100 copies/*μ*L as positive control). All reactions consisted of 40 cycles and were analyzed on a QuantStudio 3 Real-Time PCR Instrument (Applied Biosystems, Waltham, Massachusetts, United States).

### 3.2. SARS-CoV-2 IgG Serological Response

SARS-CoV-2 IgG serological response was measured using the Oracol+ sample with a salivary SARS-CoV-2 multiplex immunoassay (MIA) previously described by Pisanic et al., at Johns Hopkins University (Baltimore, Maryland) [25]. Briefly, saliva was thawed and centrifuged. Then, 10 *μ*L of supernatant was added to a 96-well microtiter plate containing 40 *μ*L assay buffer with 1000 coupled beads per bead set in each well. Each plate contained 1–2 blank wells with assay buffer instead of a sample that were used for background fluorescence subtraction. Positive controls were created by spiking SARS-CoV-2 IgG–positive saliva with high IgG levels to SARS-CoV-2 antigens into prepandemic negative saliva, used as a negative control. Assay plates were read on a Luminex MAGPIX instrument (Luminex, Austin, Texas, United States). An eight-point SARS-CoV-2 IgG standard curve of threefold serial dilutions was included on each plate in duplicate. The standard consisted of plasma from a PCR-confirmed COVID-19 sample with high SARS-CoV-2 IgG levels diluted in assay buffer. Arbitrary units (AU) ranging from 10,000 AU for the highest standard to ~5 AU for the lowest standard were assigned to each standard to assist with setting positive responses at the community samples’ level. The IgG concentration to SARS-CoV-2 antigens in AU was calculated with xPONENT Software (Luminex, Austin, Texas, United States) using a weighted five-parameter logistic curve fit.

### 3.3. Statistical Analysis

Continuous variables with skewed distributions (e.g., air concentrations) were log-transformed. Chi-square tests for categorical variables and Kruskal–Wallis tests for continuous variables were performed to assess differences in exposure and outcomes of interest across participant characteristics.

Cutoffs to discriminate salivary SARS-CoV-2 IgG positivity from negatives were created using known and confirmed negative oral fluid samples, previously described by Pisanic et al. [25]. The presence of antinucleocapsid (anti-N) IgG and antispike receptor binding domain (S-RBD) IgG was classified as indicative of the presence of prior SARS-CoV-2 infection, as was the presence of anti-S-RBD IgG alone in participants who self-reported not having received at least one COVID-19 vaccine dose. The presence of only anti-S-RBD IgG in those who self-reported receipt of at least one COVID-19 vaccine dose (regardless of vaccine type) was defined as indicative of vaccination only, whereas the absence of anti-S-RBD IgG, irrespective of anti-N IgG status, was defined as a participant who was SARS-CoV-2 viral antigen naïve. We compared these classifications to self-reported prior SARS-CoV-2 infection, self-reported COVID-19 vaccination status, and current PCR-confirmed SARS-CoV-2 infection using the saliva sample of the same participant. We built a logic tree showing the classification of IgG discrimination of participants, adapted from Duarte et al., using flowchart.fun [26].

We employed multilevel modeling grouped by household, using household exposure levels of contaminants and survey information to examine the associations between common patterns of indoor exposures by seropositive SARS-CoV-2 infection. We used logistic regression to examine binary outcomes of previous SARS-CoV-2 infection (yes/no), COVID-19 disease severity (mild/moderate–severe), and having long COVID-19 disease (yes/no), adjusting for age, sex, household income, and household size. Long COVID-19 disease was defined as the self-reported presence of persisting SARS-CoV-2 viral infection symptoms for 4 or more weeks after infection occurred [27]. We performed sensitivity analyses stratifying models by sex, vaccination status, and previous infection. All statistical analyses were performed using R software (R Version 4.4.2 [2024-10-31]). We assumed *α* = 0.05, power 80%, and two-sided tests.

## 4. Results

### 4.1. Sociodemographic and Household Characteristics

Overall, the average age of participants was 42.4 years, 60% were female, and 20% received greater than a high school education (Table 1). The older household member’s average age was 52.8 years (SD ± 12.4), and the younger household member’s average age was 31.3 years (SD ± 10.3). The median household size was four people (e.g., children; range = 2 − 14), and the median number of reported COVID-19 cases across all household members was 6 (range = 4 − 15) (Table 2). The predominant housing type for our study population was single-unit/detached homes (68%) (Table 2). Propane was the most common type of heating (70%) and cooking (71%) fuel, and electricity was the second most common for both. Smoking was never allowed in 60% of all households. Of the homes that allow smoking, the average number of reported days that someone smoked inside the home was 2.4 days/week (SD ± 3.0). Of the homes that allowed vaping, the average number of reported days that someone vaped inside the household was 0.64 days/week (SD ± 1.7).

Of the participants who were classified as positive for previous SARS-CoV-2 infection by seropositivity, 70% self-reported previously having COVID-19 disease (by confirmed PCR, rapid test, or reported symptoms). Of the participants who were classified as negative for previous infection, 59% self-reported previously having COVID-19 disease, which was marginally significantly (*p* = 0.0498) lower than the positive group. Overall, 84% of all participants reported having received at least one dose of the COVID-19 vaccine, which was not statistically different between the group that tested positive for prior SARS-CoV-2 infection versus the infection-negative group. Of the participants who previously tested positive for prior SARS-CoV-2, 24.5% self-reported long COVID-19 symptoms.

### 4.2. Evidence of SARS-CoV-2 Viral Infection

**FIGURE 1 |:**
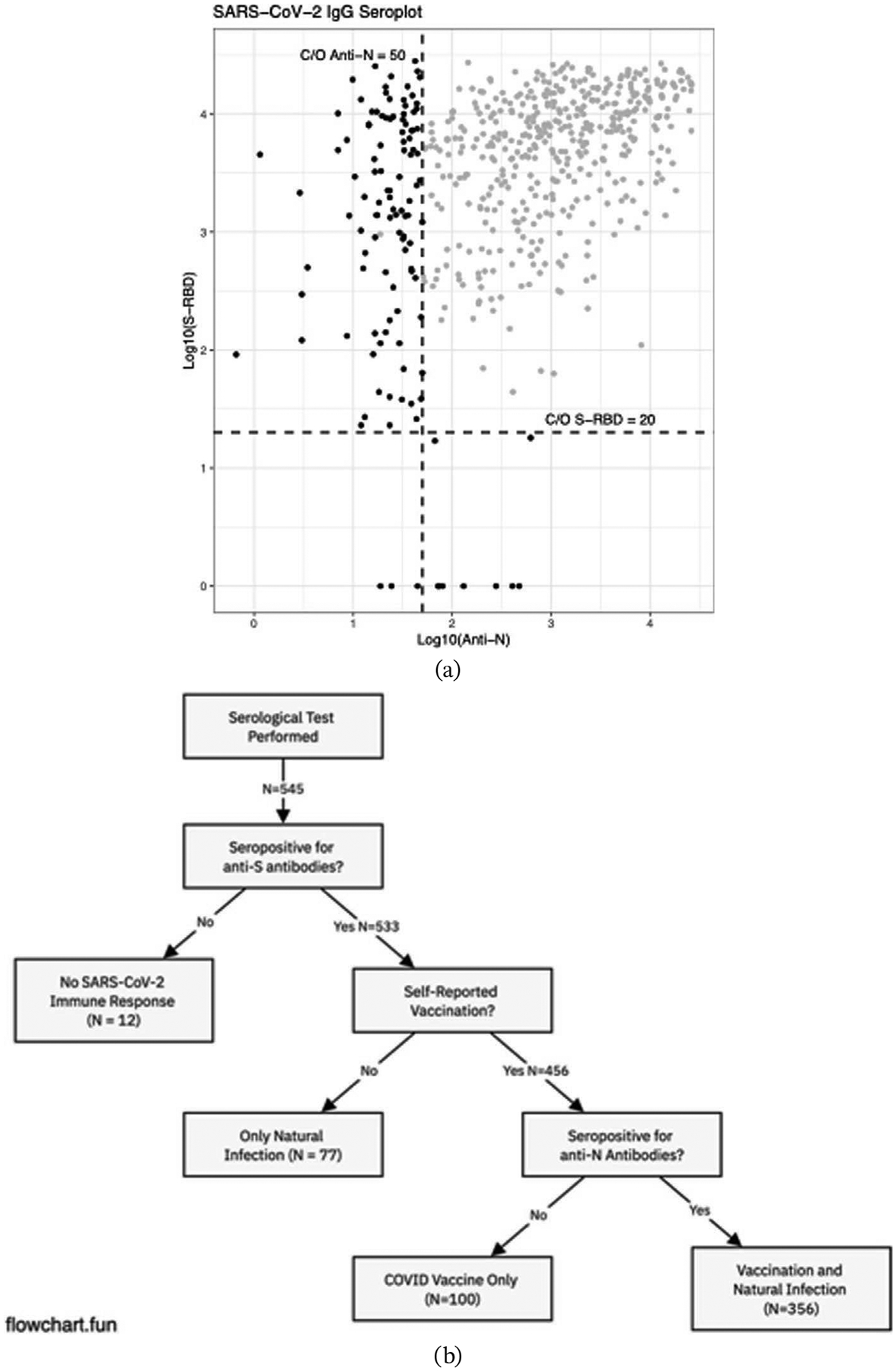
SARS-CoV-2 serological responses indicative of previous infection and/or vaccination. (a) The quadrants of the seroplot correspond to IgG classifications of SARS-CoV-2 infection and vaccination (shown in the [b] logic tree). The upper left quadrant corresponds with evidence of previous vaccination but not natural infection. The upper right quadrant (light gray points) corresponds with evidence of natural infection with or without prior vaccination. The lower left and right quadrants correspond to no prior vaccination or natural infection (SARS-CoV-2 naïve).

We identified three cases of active SARS-CoV-2 infections (RT-PCR positive) during our study recruitment period timeframe of 14 months (0.54%). Salivary testing of SARS-CoV-2 IgG analysis was successfully performed on 545 participants (out of 560 recruited) to determine previous infection (Figure 1). Evidence of previous natural infection was found in 79.4% of participants. No evidence of either vaccination or natural infection was found in 2.2% of participants (*N* = 12).

### 4.3. Indoor Air

**FIGURE 2 |:**
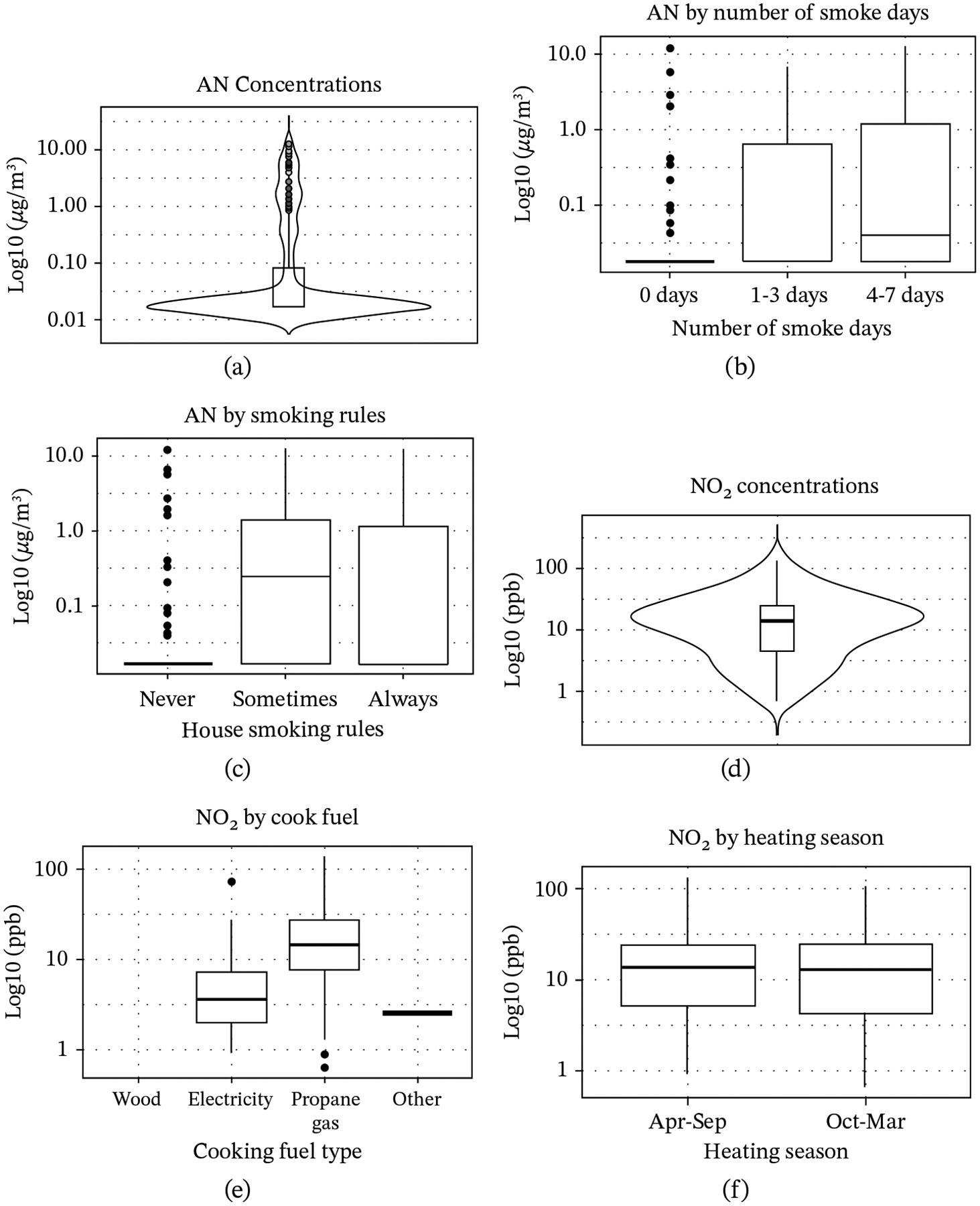
Indoor air measurements of AN and NO_2_ by self-reported household characteristics.

The AN geometric mean of all households was 0.052 *μ*g/m^3^ with a geometric standard deviation of 0.102; 59.9% of households had AN values below the LOD (Figure 2a). The concentration of AN in the household was significantly (*p* < 0.001) higher by the number of self-reported smoking days inside the home (*p* < 0.001; Figure 2b) and by reported household smoking rules (*p* < 0.001; Figure 2c). The NO_2_ geometric mean was 10.5 ppb with a geometric standard deviation of 238 (Figure 2d). The NO_2_ concentration was significantly higher in households that used propane compared to other cooking fuels (*p* < 0.001; Figure 2e). There were no significant differences between NO_2_ levels and season (Figure 2f). There were 13 households that had detectable AN despite reporting zero days of smoking inside the home (Figure 2b,c). Six of these homes had at least one inhabitant who was a current smoker; three reported vaping inside the home, but no cigarette smoking; and four were reported as nonsmoking/nonvaping.

### 4.4. Indoor Air and COVID-19 Disease Statistical Models

**FIGURE 3 |:**
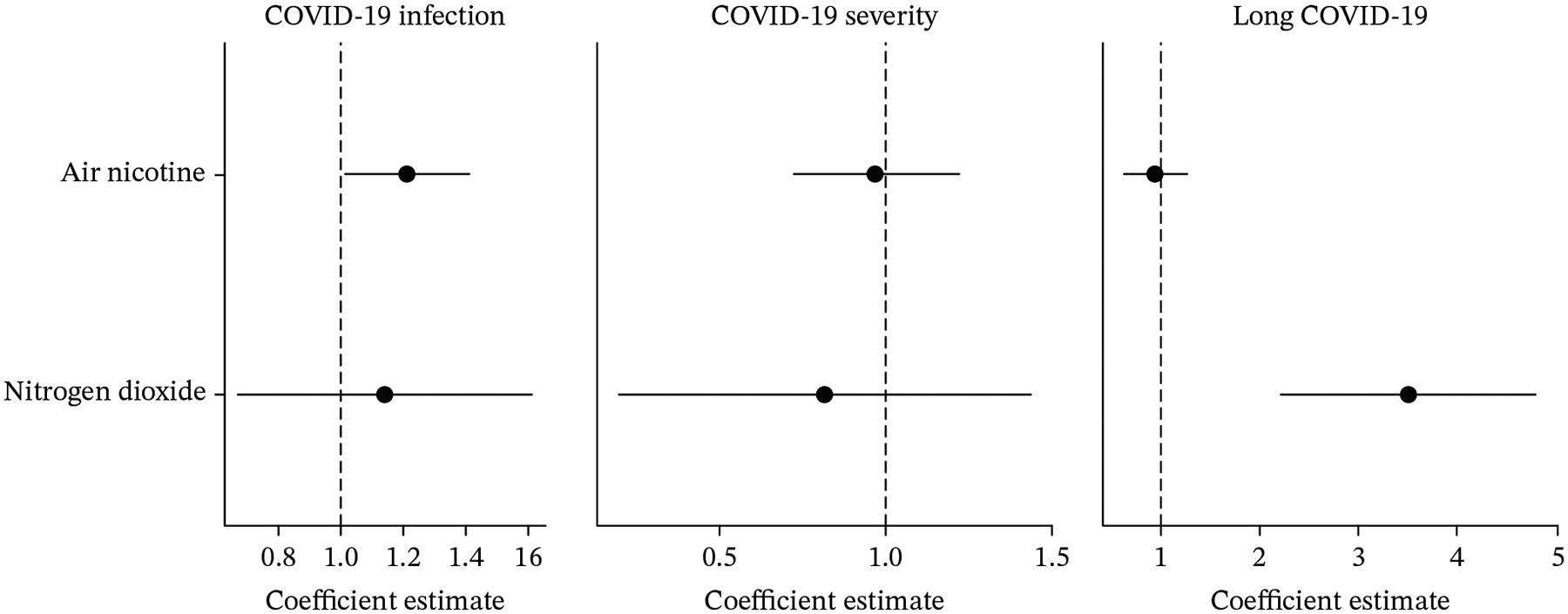
COVID-19 disease outcome odds ratios by AN and NO_2_ exposure in the COVID-19 Wayakta He? Study, 2022–2023.

The odds of prior SARS-CoV-2 viral infection were 1.21 (95% CI: 1.01, 1.51) times higher for those exposed to AN above the LOD; NO_2_ was not significantly associated with prior SARS-CoV-2 viral infection (Figure 3). Neither AN nor NO_2_ were associated with COVID-19 disease severity (mild vs. moderate/severe) (Figure 3). The odds of having long COVID-19 disease were 3.69 (95% CI: 1.11, 16.6) times higher for those exposed to NO_2_; AN was not associated with having long COVID-19 disease (Figure 3). We identified positive nonsignificant trends between salivary IgG response, increasing household income by tertile, and exposure to AN (Table S1). We did not find statistically significant relationships between serological IgG response and AN or NO_2_ in linear multilevel models (Table S1). Our sensitivity analysis found no significant associations between indoor air exposures and detectable salivary SARS-CoV-2 antigen responses or reported COVID-19 disease outcomes by gender (Tables S2 and S3).

## 5. Discussion

We investigated the association between salivary SARS-CoV-2 IgG responses (indicative of prior SARS-CoV-2 infection and/or vaccination against SARS-CoV-2) and indoor air exposures in an AI/AN community. Exposures to both AN and NO_2_ were highly prevalent in this Tribal study population. Commercial tobacco use in this population is about 50% in adults, higher than in non-Hispanic White (NHW) populations in surrounding areas [13, 14]. While previous initiatives and successful public health policies on the CRST reservation banned nicotine use in public buildings, our findings demonstrated that 40.1% of households had detectable AN levels, indicative that public health messaging needs to focus on exposures in homes as well [13, 14]. Our results align with previous research that has shown exposure to AN at home is higher among people who live in multiunit housing and have low income, both of which are highly prevalent on the CRST [28–30].

Three percent of homes exceeded the NO_2_ WHO 24-h air quality guideline of 25 *μ*g/m^3^ (80 ppb), although it is important to note that we report week-long averages, which may have had a blunting effect on NO_2_ levels [31]. Because we found no difference in NO_2_ levels by month or season, we concluded that NO_2_ levels were primarily driven by the presence of gas stoves for cooking. Current literature finds that NO_2_ exposures disproportionately affect AI/AN populations due to these communities’ reliance on alternative fuels, particularly propane for cooking [12]. Messaging about indoor NO_2_ exposures should center around proper maintenance of stoves and the use of ventilation when cooking.

We found that 78% of participants had infection-induced SARS-CoV-2 antibodies, while only 68% reported previous COVID-19 infection, likely indicative of the role of asymptomatic disease. A previous study of North Carolina households also found a high prevalence of SARS-CoV-2 seropositivity despite lower self-reported COVID-19 disease prevalence [32]. The observed under-reporting was likely influenced by the overall lack of access to primary care due to quarantine guidelines, not enough reliable SARS-CoV-2 testing opportunities, and the widely varied clinical symptoms of the disease. This highlights the importance of access to diagnostic testing and limiting exposure to others during periods of undiagnosed illness so community members can protect themselves and others in the case of future similar health crisis conditions.

Those exposed to AN were 1.21 times more likely to have antibodies indicative of SARS-CoV-2 infection. This aligns with previous studies that have found positive associations between exposure to AN and SARS-CoV-2 infection. It is possible that the SARS-CoV-2 virus can attach to AN particles in indoor environments and increase the risk of transmission [16, 33]. Additionally, we found those exposed to NO_2_ were 3.69 times more likely to report long COVID-19 disease, in line with a previous study that found that NO_2_ was associated with longer COVID-19 disease recovery, potentially due to NO_2_ impacting the immune system and increasing inflammation [17, 34]. Additionally, exposure to ambient NO_2_ has been shown to increase susceptibility to other respiratory virus infections [17, 18]. It is possible that continued exposure to NO_2_ through propane fuel for cooking in CRST households inhibited COVID-19 disease recovery and contributed to participants’ long COVID-19 disease. Our community partners at MBIRI educated us about the state of housing, as Tribal housing units are not energy-efficient. Many households put plastic sheeting or blankets over windows in the winter months to preserve heat and fuel. This could lead to decreased air flow within the homes, thus increasing indoor concentrations of NO_2_. Previous studies have also shown that public housing often has inadequate ventilation and higher levels of indoor air contaminants compared to other types of buildings [35, 36].

We did not find evidence of a predictive association between increased exposure to AN or NO_2_ and SARS-CoV-2 serological response indicative of infection. Previous studies found that exposure to ambient NO_2_ increased the IgG immune response, and exposure to cigarette smoke decreased the immune response [37–39]. This could indicate that in our population, the week-long exposure assessment may not properly reflect true, chronic indoor air exposures to establish associations with SARS-CoV-2 viral infection outcomes. Historically, AI/AN populations have been mistreated and discriminated against by the US government, which has led to many health disparities among Native communities [40–42]. In a previous analysis by our team, we found that this population likely underreported chronic health outcomes that are otherwise known to be highly prevalent in the AI/AN community [14]. It is possible that lack of access to healthcare and having risk factors for adverse COVID-19 disease outcomes are driving the COVID-19 serological response rather than solely indoor air toxicant exposures.

Those in higher income categories in our study had significantly higher SARS-CoV-2 positivity compared to those in the lowest income category. This does not align with other studies that found that those with lower income were a predictor of having SARS-CoV-2 infections, often due to the inability to work from home [43, 44]. The CRST’s 2022 median household income of $54,495 was substantially lower than the US median of $74,580, with a poverty rate (34.2%) that is more than triple the US average (11.5%) [30, 45, 46]. During the pandemic, many CRST Tribal members were out of work, whereas those in the highest income category were more likely to be exposed to the virus via work. These findings highlight the importance of conducting culturally relevant, community-driven research in AI/AN communities, as the intricacies of social life and circumstances need to be considered respectfully during data collection and analyses. Due to the wide range of cultural, linguistic, and social structures reflected among all AI/AN Tribes and Nations, data collection and health research cannot force previously thought social and/or scientific constructs on the communities. This approach has been beneficial when our team investigated these unusual SARS-CoV-2 infection trends and COVID-19 disease outcomes that do not parallel those previously described in NHW populations.

There are several limitations to this study. While we assessed serological response to SARS-CoV-2 infection, we recruited households over a long period of time, which encompassed many different waves of the pandemic. Some participants who were recruited later in the study, who tested negative for previous infection, may have been infected very early on in the pandemic, and their SARS-CoV-2 IgG levels had waned below the cutoff by the time of sampling [47]. Indoor air sampling limited to 7 days (1 week) may not accurately portray chronic AN or NO_2_ levels and may have introduced exposure misclassification. Additionally, we were limited to measuring NO_2_ in only 100 homes of our population, which reduced power in some of our models and affected generalizability. Finally, we relied on self-reported questionnaire data for household characteristics, which may have reduced the accuracy of some models due to the possibility of recall bias.

Notwithstanding, our study has many strengths, as this is the first study that was carried out during the midst of the COVID-19 pandemic with strong Tribal leadership support, as we established indoor toxicant exposures in households and salivary SARS-CoV-2 infection associations. Our recruitment of households on the CRST reservation encompassed 21 out of the total 23 communities within the reservation and contributed to our large sample size as well as community representativeness of our sample. Additionally, we collected direct measurements of AN, NO_2_, and salivary biomarkers. Although this study focused solely on indoor air pollution and its health effects in Tribal households, we see future opportunities in applying more methods, such as GIS and remote sensing works, that would support community-driven and community-based research concerns (e.g., wildfires) in air pollution monitoring, as shown successfully by others [48, 49].

This study found that exposure to AN increased the odds of COVID-19 disease, and exposure to NO_2_ increased the odds of long COVID-19 disease symptoms. Additionally, we found that in this rural Native population, higher income was associated with increased likelihood of SARS-CoV-2 infection. This information is valuable to our community partners in the CRST to evaluate and change the necessary community health response and future pandemic preparedness. Actionable items such as improving indoor ventilation, providing access to low-cost air filtration, improving public health messaging surrounding secondhand smoke, and providing smoking cessation resources could be recommended. Public health messaging on the CRST should focus on increasing awareness of how indoor air environments can impact critical and severe disease and infectious health outcomes and highlight education on how to reduce exposures in Tribal households.

## Supplementary Material

Supplementary Material

Additional supporting information can be found online in the Supporting Information section. (Supporting Information) Table S1. SARS-CoV-2 Gen_N Response (All Participants). Table S2. SARS-CoV-2 Gen_N Response stratified by Gender. Table S3. COVID-19 infection outcome odds ratios stratified by gender.

## Figures and Tables

**TABLE 1 |:** Sociodemographic characteristics of the CRST COVID-19 Wayakta He? Study participants by previous SARS-CoV-2 infection (*N* = 546). Stratified by positivity for previous SARS-CoV-2 infection, identified by serological response. Self-reported COVID-19 was reported by participants and can include a PCR-confirmed test, an at-home rapid test positive, or symptoms. Chi-square tests for categorical variables and Kruskal–Wallis tests for continuous variables were performed.

	Overall	Positive	Negative	
Characteristic	*N* = 546	*N* = 428	*N* = 118	*p* value
Age, mean (SD)	42.4 (15.7)	41.8 (15.5)	44.9 (16.6)	0.07
Gender				0.64
Male	40.0%	40.0%	44.0%	
Female	60.0%	60.0%	56.0%	
Education				0.13
< High school	20.0%	19.0%	21.0%	
High school/GED	60.0%	59.0%	65.0%	
≥ High school	20.0%	22.0%	14.0%	
Self-reported COVID-19	68.0%	70.0%	59.0%	0.05
Vaccination rates				0.50
At least 1 dose	83.9%	83.1%	87.3%	
No doses	16.1%	16.9%	12.7%	

**TABLE 2 |:** Characteristics of the CRST COVID-19 Wayakta He? Study households by number of household COVID-19 cases (*N* = 253). Households were stratified by the median number of reported COVID-19 cases across all household members. Chi-square tests for categorical variables and Kruskal–Wallis tests for continuous variables were performed.

	Overall	Low household *COVID* – 19 ≤ 6	High household *COVID* – 19 > 6	
Characteristic	*N* = 253	*N* = 118	*N* = 135	*p* value
Household size, mean (SD, range)	4.80 (2.97, 2–33)	4.34 (2.24, 2–14)	4.00 (2.97, 2–14)	0.016
Household income, % (*n*)				0.31
$0–$2000 monthly	56.7 (115)	51.8 (57)	62.4 (58)	
$2001–$5000 monthly	24.1 (49)	27.3 (30)	20.4 (19)	
$5001+ monthly	19.2 (39)	20.9 (23)	17.2 (16)	
Household smoking rules, % (*n*)				0.28
Allowed	12.4 (31)	9.7 (13)	15.4 (18)	
Sometimes allowed	25.9 (65)	24.6 (33)	27.4 (32)	
Never allowed	61.7 (155)	65.7 (88)	57.2 (67)	
Household vaping rules, % (*n*)				0.34
Allowed	9.7 (24)	7.6 (10)	12.1 (14)	
Sometimes allowed	18.1 (45)	16.7 (22)	19.8 (23)	
Never allowed	72.2 (179)	75.7 (100)	68.1 (79)	
Type of heating fuel, % (*n*)				0.84
Propane	69.6 (176)	71.1 (96)	67.8 (80)	
Electricity	24.9 (63)	23.0 (31)	27.1 (32)	
Other	5.5 (14)	5.9 (8)	5.1 (6)	
Type of cooking fuel, % (*n*)				0.99
Propane	70.2 (177)	69.4 (93)	71.2 (84)	
Electricity	28.2 (71)	29.1 (39)	27.1 (32)	
Other	1.6 (4)	1.5 (2)	1.7 (2)	
Type of housing, % (*n*)				0.055
Single unit/detached	69.8 (176)	76.3 (103)	62.4 (73)	
Multiunit/apartment	23.0 (58)	17.8 (24)	29.1 (34)	
Shelter/no consistent home	7.2 (18)	5.9 (8)	8.5 (10)	
Number of smoke days in the last week, mean (SD)	2.42 (3.00)	2.19 (1.49)	2.68 (3.09)	0.19
Number of vape days in the last week, mean (SD)	0.64 (1.7)	0.48 (1.5)	0.83 (1.9)	0.11

## Data Availability

All data generated by this grant are owned by the Cheyenne River Sioux Tribe as described by the Cheyenne River Sioux Tribe Data Use Policy (May 2020), and access to the data, metadata, or results is subject to institutional board and Tribal Council approval. These processes can be accessed and discussed with Drs. Tillery and Erdei.

## Summary:

- Tribal communities have a high prevalence of exposure to air nicotine (AN) and nitrogen dioxide (NO_2_).
- Tribal communities have higher odds of COVID-19 infection with exposure to AN in homes.
- Tribal communities have higher odds of having long COVID-19 disease with exposure to NO_2_.
- This information is valuable to public health preparedness in rural communities.
