# Supplementary material for: Indoor Air Nicotine, Nitrogen Dioxide Exposure, and COVID-19 Outcomes in a Great Plains Tribal Community
Source: Indoor Air. Author manuscript; Available in PMC 2026 Aug 8. (PMC13451034; doi:10.1155/ina/7749725)
Supplement: Supplementary file 1 [file NIHMS2200448-supplement-Supplementary_Material.docx]

**Supplemental Material**

Table S1. SARS-CoV-2 Gen_N Response (All Participants)

| **Coefficient** | **AN** | **NO2** |
| --- | --- | --- |
| Unadjusted | 0.970  (0.846, 1.11) | 0.908  (0.662, 1.24) |
| Adjusted | 1.03  (0.936, 1.11) | 0.993  (0.848, 1.16) |
| Age | 0.991  (0.984, 0.998) | 0.996  (0.986, 1.01) |
| Gender | 0.964  (0.768, 1.21) | 0.923  (0.666, 1.28) |
| HH Income 2:1 | 1.33  (0.928, 1.90) | 0.944  (0.615, 1.45) |
| HH Income 3:1 | 1.31  (0.889, 1.93) | 1.18  (0.751, 1.87) |
| HH Size | 1.01  (0.969, 1.05) | 0.997  (0.914, 1.09) |

Table S2. SARS-CoV-2 Gen_N Response stratified by Gender

| Model | Men | | Women | |
| --- | --- | --- | --- | --- |
|  | AN | NO2 | AN | NO2 |
| Unadj. | 0.943 (0.791, 1.13) | 0.948 (0.609, 1.48) | 1.00 (0.835, 1.20) | 0.901 (0.617, 1.32) |
| Adj. | 1.04 (0.830, 1.30) | 1.14 (0.654, 1.98) | 1.14 (0.895, 1.44) | 0.937 (0.602, 1.46) |

Table S3. COVID-19 infection outcome odds ratios stratified by gender

| Variable | Model | Men | | Women | |
| --- | --- | --- | --- | --- | --- |
|  |  | AN | NO2 | AN | NO2 |
| COVID-19 Infection | Unadj. | 1.02 (0.856, 1.23) | 0.701 (0.371, 1.22) | 1.00  (0.836, 1.22) | 1.10 (0.688, 1.74) |
|  | Adj. | 1.24 (0.967, 1.66) | 1.15 (0.396, 3.50) | 1.25  (0.939, 1.84) | 1.16 (0.674, 2.02) |
| COVID-19 Severity | Unadj. | 0.813 (0.470, 1.15) | 0.988 (0.504, 2.07) | 0.959 (0.703, 1.22) | 1.59 (0.853, 3.37) |
|  | Adj. | 0.746 (0.417, 1.12) | 0.0830 (0.00045, 0.745) | 1.15  (0.805, 1.55) | 1.22 (0.540, 3.29) |
| Long COVID | Unadj. | 0.899  (0.680, 1.14) | 0.840  (0.502, 1.38) | 1.13  (0.897, 1.43) | 0.972  (0.594, 1.63) |
|  | Adj. | 0.751  (0.437, 1.06) | 1.83 (0.743, 5.40) | 0.969 (0.654, 1.34) | 1.15 (0.606, 2.25) |
